# Iatrogenic aortic dissection during aortic root replacement in an older Loeys–Dietz syndrome type III patient with no family history of aortic disease: a case report

**DOI:** 10.1186/s13019-023-02430-y

**Published:** 2023-11-08

**Authors:** Kenichi Kato, Ken Nakamura, Kaho Kato, Shusuke Arai, Shuto Hirooka, Cholsu Kim, Hideaki Uchino, Takao Shimanuki

**Affiliations:** 1 Division of Cardiovascular Surgery, Nihonkai General Hospital, 30 Akiho-cho, Sakata, Yamagata 998-8501 Japan; 2 Certified Genetic Counselor, Nihonkai General Hospital, Sakata, Japan

**Keywords:** Iatrogenic aortic dissection, Loeys–Dietz syndrome, *SMAD3* mutation, Connective tissue disease in older patients

## Abstract

**Background:**

Iatrogenic aortic dissection during cardiac surgery is a rare but critical complication. At present, no strategies have been developed to prevent it. We herein report a case of intraoperative aortic dissection during aortic root replacement in an older patient with Loeys–Dietz syndrome type III who had no family history of aortic disease.

**Case presentation:**

A 60-year-old man was admitted to the hospital for Stanford type B acute aortic dissection and given conservative treatment. He was found to have aortic root dilatation and severe aortic regurgitation. Thus, elective Bentall procedure was performed. Postoperative computed tomography showed new Stanford type A aortic dissection that may have developed due to aortic cannulation during surgery. The patient was given conservative treatment and successfully discharged to home at postoperative day 34. Although he had no family history of aortic disease, a genetic test revealed an unreported *SMAD3* frameshift mutation (c.742_749dup, p. Gln252ThrfsTer7), and the patient was diagnosed with Loeys–Dietz syndrome type III.

**Conclusion:**

In patients with connective tissue disorder, aortic manipulations may become the cause of critical complications. Avoiding the use of invasive techniques, such as cannulation and cross-clamping, and implementing treatment strategies, such as perfusion from other sites than the aorta and open distal anastomosis, can prevent these complications, and may be useful treatment modalities. The possibility of connective tissue disease should be considered even if the patient is older and has no family history of aortic disease.

## Background

Iatrogenic aortic dissection (IAD) during cardiac surgery is a rare complication, with a reported incidence rate of 0.04–0.23% [1–5]. However, it is potentially fatal, with a reported operative mortality rate of as high as 35.5–48% [4–6]. Although previous studies proposed strategies to prevent it [6, 7], no measures have been established to date. We herein report a case of intraoperative Stanford type A aortic dissection during aortic root replacement in an older patient with Loeys–Dietz syndrome (LDS) type III with an unreported pathogenic *SMAD3* mutation who had no family history of aortic disease.

## Case presentation

**Fig. 1:**
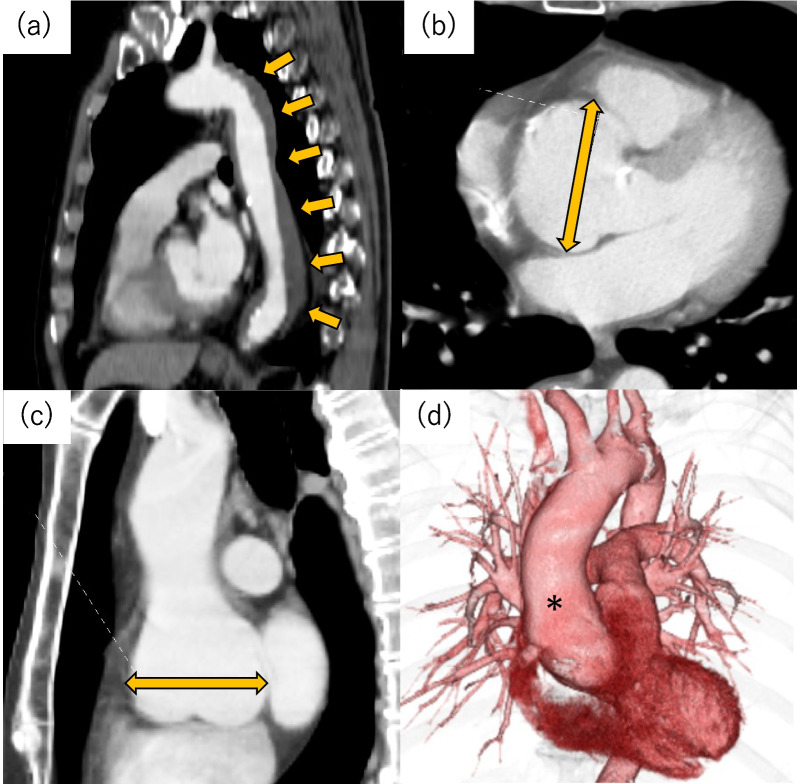
Uncomplicated Stanford type B, DeBakey type IIIb acute aortic dissection with thrombosed false lumen 14 months before surgery. Aortic root dilatation was also detected. **a** Stanford type B acute aortic dissection (arrows), aortic root dilatation of 55 mm in **b** axial and **c** sagittal images (arrows), **d** aortic root dilatation (asterisk) in 3D-CT angiography.

**Fig. 2:**
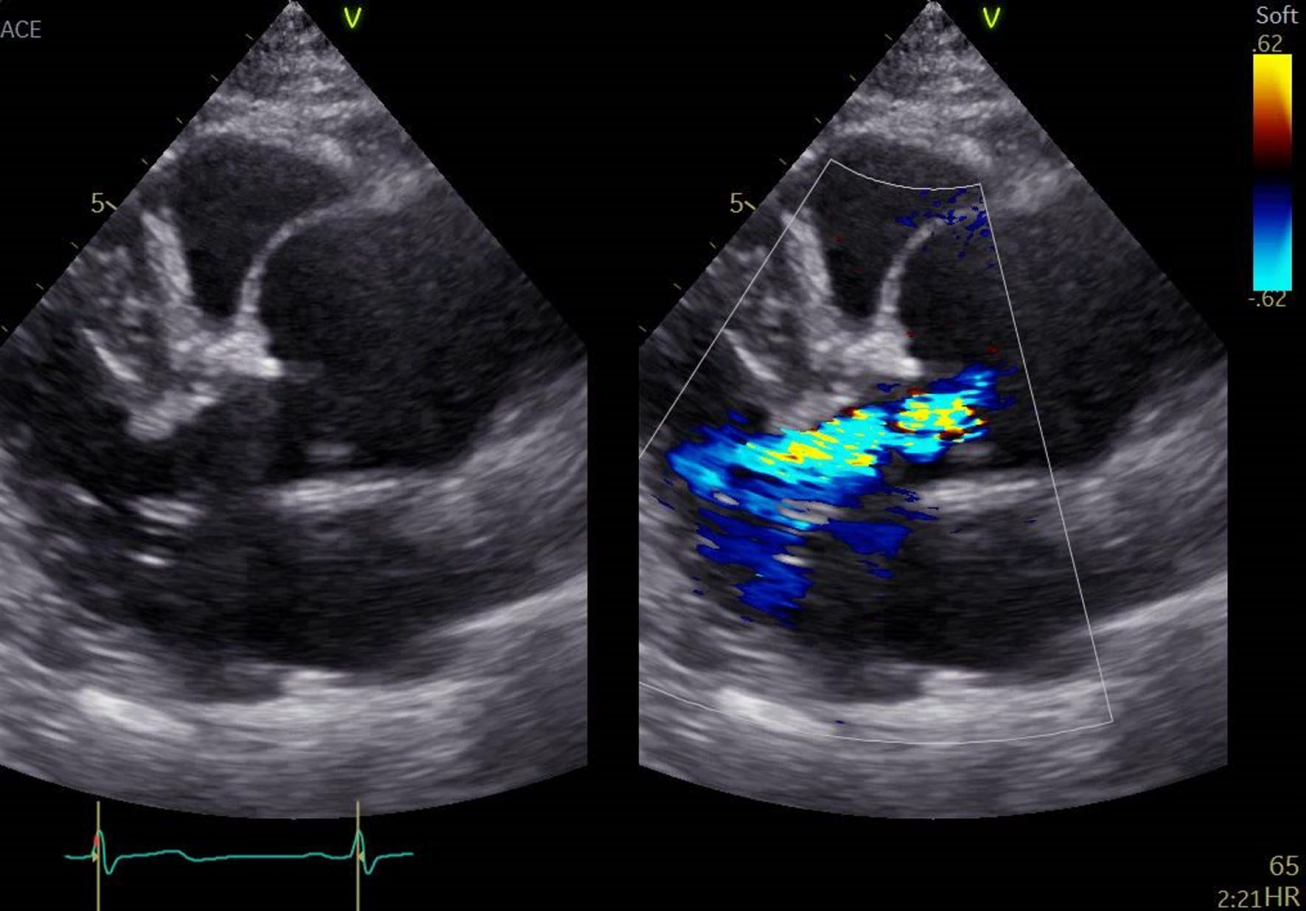
Aortic regurgitation due to annuloaortic ectasia on transthoracic echocardiogram

A 60-year-old man presented to the hospital with back pain and was subsequently diagnosed with uncomplicated Stanford type B acute aortic dissection. He was hospitalized and given conservative treatment; however, a contrast-enhanced computed tomography (CT) scan at that time showed a 55-mm aortic root dilatation (Fig. 1). Furthermore, transthoracic echocardiogram revealed severe aortic regurgitation (AR), and the patient was diagnosed with annuloaortic ectasia (Fig. 2). Its vena contracta was 6 mm, and the pressure halt time was 430 ms. Holodiastolic flow reversal was also detected in the abdominal aorta. Cusp degeneration and mitral valve insufficiency were not observed. After discharge, false lumen stability was confirmed on follow-up CT; thus, elective surgery for the aortic root was scheduled. The patient’s other significant past medical history included hypertension, dyslipidemia, hyperuricemia, sleep apnea, and vasospastic angina, which developed 11 months before. He was 183-cm tall and weighed 83.0 kg and had no specific family history.

During surgery, cardiopulmonary bypass (CPB) was established via cannulation to the ascending aorta and the right atrium using a 24 Fr curved-tip dispersion aortic cannula and a 34/46 Fr two-stage venous cannula, respectively. In addition, an antegrade cardioplegia (CP) cannula was cannulated to the proximal ascending aorta, and then cardiac arrest was induced with antegrade CP after cross-clamping of the ascending aorta. Aside from a small calcification at the nadir of the annulus of the right coronary cusp, aortic valve degeneration such as cusp size discrepancy, cusp thickening, or cusp fenestration was not observed; thus, the David procedure was initially performed. However, intraoperative transesophageal echocardiogram revealed eccentric AR jet due to right coronary cusp falling toward the left ventricle. Therefore, valve sparing was abandoned, and the Bentall procedure using a mechanical valve was selected. The antegrade CP cannulation site was resected and replaced with vascular prosthesis. Smooth CPB weaning was achieved, and the procedure was completed without any problems. The operation and CPB times were 416 and 308 min, respectively.

**Fig. 3:**
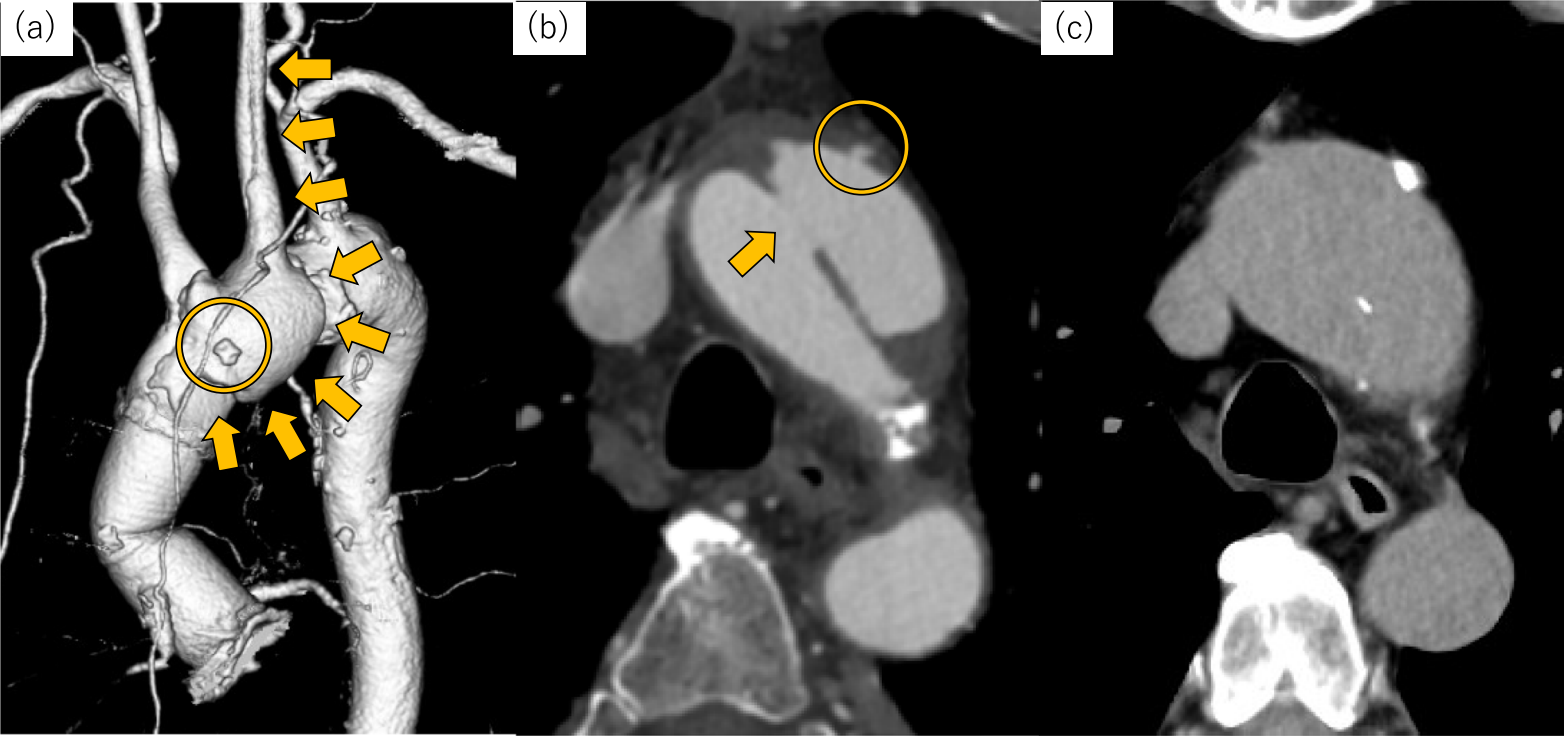
Contrast-enhanced CT at POD 14 (**a**, **b**) and plain CT at 6 months after surgery (**c**). **a** Postoperative Stanford type A aortic dissection (arrows). Dissection was extended to the left common carotid artery. Felt pledget is located at the aortic cannulation site (circle). The aortic root had been replaced (asterisk). **b** The entry was at the distal ascending aorta (arrow) just below the felt pledget at the cannulation site (circle). **c** The aortic diameter was not changed

The postoperative course was not complicated, and the patient had never complained of chest pain after the operation. However, a routine contrast-enhanced CT scan at postoperative day (POD) 14 showed new Stanford type A aortic dissection (Fig. 3). It was thought to be caused by intraoperative aortic cannulation for CPB as the entry was just below the felt pledget used for aortic cannulation site closure. The postoperative dissection was conservatively treated as the aortic root had already been replaced, and no symptoms, or malperfusion was observed. The patient was carefully observed with continuing hospitalization, and the resting level was gradually eased. Follow-up CT at POD 29 showed no enlargement of the ascending aorta, and the patient was discharged without other complications at POD 34.

**Fig. 4:**
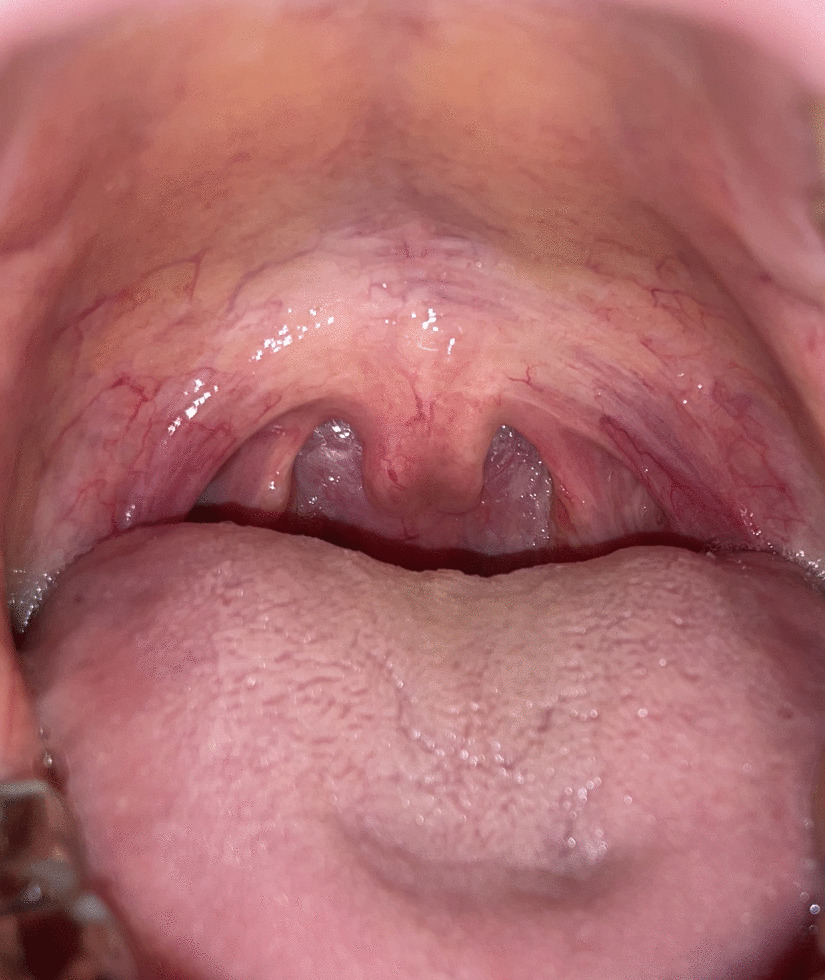
Bifid uvula (cleft palate was not observed)

Although he had no family history of cardiovascular diseases, including aortic dissection, or aortic aneurysm, connective tissue disease was suspected due to his own history of aortic root dilatation and recurrent aortic dissection; thus, a genetic test was conducted (tested genes: *FBN1*, *FBN2*, *TGFBR1*, *TGFBR2*, *TGFB2*, *TGFB3*, *SMAD2*, *SMAD3*, *ACTA2*, *COL3A1*, *EFEMP2*, *FLNA*, *MYH11*, *MYLK*, *SLC2A10*). Among the tested genes, unreported heterozygous 8-base duplication mutation in the *SMAD3 MH2* domain (c.742_749dup, p. Gln252ThrfsTer7) and likely benign heterozygous missense mutation in *FBN2* (c.3518C > G, p. Thr1173Ser) were detected [8]. The SMAD3 mutation seemed to be pathogenic because it causes frameshift, and premature termination codon can be formed. When the oropharynx was examined, a bifid uvula was found (Fig. 4). Although the patient had no history of musculoskeletal disease, such as osteoarthritis, he was diagnosed with LDS type III based on the diagnostic criterion [9].

The aortic diameter was unchanged on follow-up CT at 6 months after surgery (Fig. 3); thus, observation was continued. If aortic arch enlargement is seen in the future, surgical treatment such as total arch replacement with frozen elephant trunk will be planned.

## Discussion

Although IAD during cardiovascular surgery is rare [1–5], there have been several reports of IAD in connective tissue diseases. *Yoneyama* et al. reported the case of a patient with LDS type III with *MYH11* co-mutation who developed intraoperative Stanford type A aortic dissection immediately after CPB initiation [10]. Cases of IAD during cardiovascular surgery in patients with Marfan syndrome have also been reported [7, 11, 12]. Patients with connective tissue disease are at a high risk of developing IAD. Unfortunately, there are currently no established strategies to prevent IAD in such patients. *Kumar* et al. demonstrated that using a curved-tip dispersion cannula could minimize the risk of dissection [7]. It may prevent lesser curvature intimal tear than the use of a straight-tip cannula but may not prevent dissection from the cannulation site similar to this case. CPB with perfusion to other sites, such as common femoral artery (CFA) and axillary artery (AxA), instead of the ascending aorta may be effective for protecting the aorta in such patients. Furthermore, perfusion through a prosthetic vascular graft anastomosed to the CFA and/or AxA may be more protective if arterial dissection due to cannulation is a concern. In addition, aortic cross-clamping can also cause IAD [3]. To prevent it, *Von Aspern* et al. suggested the use of padded aortic cross-clamps or cross-clamps that generate less force [6]. However, nonuse of aortic cross-clamping, if possible, seems to be more protective. Open-distal anastomosis under hypothermic circulatory arrest can avoid the use of aortic cross-clamping and may be considered for cases of connective tissue disease. However, this needs further investigation.

The patient described here developed Stanford type B acute aortic dissection and was also found to have aortic root dilatation at 60 years old. *Jannuzi* et al. reported that the mean age of aortic dissection onset in Marfan syndrome is 35 years, and Loeys et al. reported that the mean ages at first major event (vascular surgery, dissection, or death) in LDS types I and II are 24.5 and 29.8 years, respectively [13, 14]. Pepin et al. also reported that the mean age at first complication (arterial rupture, dissection, aneurysm, etc.) among patients with vascular Ehlers–Danlos syndrome is 23.5 years, and more than 80% of them experience complication by the age of 40 years [15]. Compared with them, the onset seems to be later in the present case. Furthermore, *Hostetler* et al. reported that the median age at first aortic event (dissection, rupture, or elective aortic aneurysm repair) among patients with pathogenic *SMAD3* mutation is 47 years, which is later compared with patients with *TGFBR1* and *TGFBR2* mutations [16]. They also reported that patients with haploinsufficiency mutation in the *SMAD3 MH2* domain are older at first aortic event than those with *MH2* missense mutation　(median age: 49 vs. 42 years) [16]. The *MH2* domain mutation in the present case is categorized as a haploinsufficiency variant due to the early formation of stop codons. The relatively late onset of aortic event in this patient can be explained by his genetic feature. However, it may be difficult to suspect connective tissue disease if the age of first aortic event is older, especially that there is no family history of aortic disease like this patient. If connective tissue disease is not suspected, no precaution can be taken to prevent IAD during surgery. We think that it is important to consider connective tissue disease when multiple aortic diseases exist, like the present case, even if the patient is older. Furthermore, it is likely that patients with connective tissue disease diagnosed at an older age often have children. Inheritance and risk management for the next generation should also be considered.

The patient had an unreported heterozygous frameshift mutation due to 8-base duplication in the *SMAD3 MH2* domain (c.742_749dup, p. Gln252ThrfsTer7). This *SMAD3* mutation is not registered in ClinVar [8]; however, it seems to be pathogenic as it is expected to be a loss-of-function mutation by forming a premature termination codon that causes nonsense-mediated mRNA decay. *MacCarrick* et al. reported that a mutation in any of the LDS-associated genes including *SMAD3* in combination with documented aneurysm or dissection is sufficient to diagnose LDS [9]. Based on this criterion, the patient in the present case can be diagnosed with LDS type III. About 75% of LDS type III cases are caused by de novo* SMAD3* mutation [17]. The mutation of the patient in this case was also thought to be de novo as he did not have a family history of cardiovascular diseases and sudden deaths.

Among his three children, the eldest (30-year-old woman) had the same *SMAD3* mutation. She was 178-cm tall and weighed 56.3 kg. Furthermore, she had bifid uvula but no history of musculoskeletal disease. Contrast-enhanced CT and transthoracic echocardiogram did not reveal aortic root dilatation, AR, or aortic dissection. We planned to make a follow-up on her and the patient, her father.

## Conclusion

We experienced IAD in a patient with LDS type III with novel *SMAD3* mutation (c.742_749dup, p. Gln252ThrfsTer7). The IAD was suspected to have been caused by aortic cannulation. To minimize IAD, the use of surgical strategies such as AxA and CFA perfusion plus open-distal anastomosis may be considered, particularly for patients with connective tissue disease. The possibility of connective tissue disease should be considered even if the patient is older and has no family history of aortic disease.

## Data Availability

The datasets used during the current report are available from the corresponding author upon reasonable request.

## Abbreviations

IAD: Iatrogenic aortic dissection
LDS: Loeys–Dietz syndrome
CT: Computed tomography
AR: Aortic regurgitation
CPB: Cardiopulmonary bypass
NMD: Nonsense-mediated mRNA decay
CFA: Common femoral artery
AxA: Axillary artery
